# Exploring the nature of calls to South African mental health helplines

**DOI:** 10.4102/sajpsychiatry.v24i0.1279

**Published:** 2018-09-26

**Authors:** Dessy Deysel, Linda E. Blokland

**Affiliations:** 1 Department of Psychology, University of Pretoria, South Africa

## Abstract

**Introduction:**

In South Africa, 30.3% of the population experience mental illness during their lifetime. At least 1 in 10 non-natural deaths are because of suicide, and poverty, crime and HIV are common local psychosocial stressors. Despite this, it is estimated that only 5.0% of the national health expenditure is allocated to mental health, and nearly 75.0% of those with a mental health condition are untreated. Within this context exists an NPO offering free national mental health helplines, yet little is known about the population accessing these lines, the nature of difficulties reported and the kind of assistance provided. It is this knowledge gap this study addresses.

**Methods:**

During this exploratory mixed-method archival research, the NPO’s existing telephone counselling records from February and March 2017 were sampled (*N* = 300) to determine caller demographics, timing of calls, object of concern, nature of difficulty expressed and form of assistance. Descriptive, inferential and content analyses were applied to evaluate the data.

**Results:**

Results show that helpline callers were mostly female (76.3%), aged 20 to 29 years (41.7%), of black race (61.3%) and from Gauteng (56.0%). The highest volume of calls was received on Thursdays (23.0%), and the busiest time was the 12:00 to 16:00 shift (47.3%). The majority of people called for self-related concerns (85.0%), most frequently mental illness (227 references) and interpersonal problems (105 references). The NPO predominantly assisted callers with referrals to other resources (86.0%), mainly support structures (144 references) and counselling services (127 references) run by other NPOs. The discovery of significant associations between age and race, gender and race, province and race, age and week day, province and shift, week day and shift suggests that the caller profile may vary depending on race and temporal factors.

**Conclusion:**

Callers who most readily reached out to the helplines were women, and those aged between 20 and 40 years. Mental health support and counselling services appeared to be most needed, with a heavy reliance on NPOs – at least double that on government services, and nearly triple that on the private sector. This suggests that informal resources are central to South Africa’s mental health care system, substantiating greater investment in informal community care.

